# The Interactions between Host Glycobiology, Bacterial Microbiota, and Viruses in the Gut

**DOI:** 10.3390/v10020096

**Published:** 2018-02-24

**Authors:** Vicente Monedero, Javier Buesa, Jesús Rodríguez-Díaz

**Affiliations:** 1 Department of Food Biotechnology, Institute of Agrochemistry and Food Technology (IATA, CSIC), Av Catedrático Agustín Escardino, 7, 46980 Paterna, Spain; btcmon@iata.csic.es; 2 Departament of Microbiology, Faculty of Medicine, University of Valencia, Av. Blasco Ibañez 17, 46010 Valencia, Spain; Javier.Buesa@uv.es

**Keywords:** rotavirus, norovirus, secretor, *fucosyltransferase-2* gene (FUT2), histo-blood group antigens (HBGAs), microbiota, host susceptibility

## Abstract

Rotavirus (RV) and norovirus (NoV) are the major etiological agents of viral acute gastroenteritis worldwide. Host genetic factors, the histo-blood group antigens (HBGA), are associated with RV and NoV susceptibility and recent findings additionally point to HBGA as a factor modulating the intestinal microbial composition. In vitro and in vivo experiments in animal models established that the microbiota enhances RV and NoV infection, uncovering a triangular interplay between RV and NoV, host glycobiology, and the intestinal microbiota that ultimately influences viral infectivity. Studies on the microbiota composition in individuals displaying different RV and NoV susceptibilities allowed the identification of potential bacterial biomarkers, although mechanistic data on the virus–host–microbiota relation are still needed. The identification of the bacterial and HBGA interactions that are exploited by RV and NoV would place the intestinal microbiota as a new target for alternative therapies aimed at preventing and treating viral gastroenteritis.

## 1. The Relevance of the Enteric Viruses Rotavirus and Norovirus

According to the World Health Organization (WHO), diarrheal disease is the second leading cause of death in children under five years, provoking around 525,000 deaths each year [1]. Acute gastroenteritis (AGE) is caused by a variety of pathogens including parasites, bacteria, and enteric viruses. In 2015, rotavirus (RV) infections were the leading cause of deaths due to AGE in children under the age of five (146,000 deaths, 118,000–183,000) [2]. RV infections in humans occur throughout their lives but the resulting disease is mild and often asymptomatic [3]. In addition to sporadic cases of acute gastroenteritis, outbreaks of RV diarrhea in school-aged children and adults have increasingly been reported [4,5]. RV has been known to produce disease in humans since 1973 [6,7]. The main goal in the fight against RV infection has been the development of RV vaccines. Since the 80s, this was the focus of RV research and since 2006 two vaccines (Rotateq^TM^ and Rotarix^TM^) have been licensed in many countries around the world. The genus *Rotavirus* belongs to the *Reoviridae* family and their viral particles show icosahedral symmetry consisting of three concentric protein layers with ~100 nm in diameter, including the spikes. The viral genome is composed of 11 segments of double-stranded RNA and codes for 12 proteins, 6 structural proteins (VP1, VP2, VP3, VP4, VP6, VP7) and 6 non-structural proteins (NSP1 to NSP6). Each RNA segment contains a single open reading frame (ORF) except the segment eleven that codes for two proteins (NSP5 and NSP6) [8]. RV are classified into at least 7 groups (A to G) according to the immunological reactivity of the VP6 middle layer protein, with group A RV being the most commonly associated with infections in humans [8]. A classification system of group A RV into G (depending on VP7, a glycoprotein) and P (from the VP4 protein, that is sensitive to proteases) types has been established. So far, 35 G-genotypes and 50 P-genotypes have been identified among human and animal RV, depending on VP7 and VP4, respectively [9]. Viruses carrying G1P[8], G2P[4], G3P[8] and G4P[8] represent over 90% of human RV strains co-circulating in most countries, although other G and P combinations are being isolated in increasing numbers [8]. The existing vaccines, indeed, protect against these genotypes. Rotateq^TM^ is a tetravalent vaccine (G1 to G4 with a P[8]) while Rotarix^TM^ is a monovalent (only G1P[8]) vaccine.

After the introduction of RV vaccines, norovirus (NoV) has emerged as the leading cause of diarrhea in the pediatric population in some areas of the world [10,11]. NoV are also the leading cause of foodborne outbreaks of AGE with an estimate of 120 million cases in 2010 [12]. Contrarily to RV, no vaccines are commercialized for NoV so far. These viruses are ubiquitous and associated with 18% (95% CI: 17–20%) of diarrheal disease cases globally, with similar proportions of disease in high-, middle- and low-income settings. NoV AGE has high social costs and it is estimated to cause approximately 200,000 deaths annually worldwide, with 70,000 or more among children in developing countries [13]. Furthermore, NoV AGE also derives in high economic costs as a result of hospitalizations and work absenteeism. Knowledge on the human NoV (hNoV) pathobiology was hampered until recently by the lack of a system to reproduce viral infection in vitro [14]. NoV are non-enveloped viruses with a T3 icosahedral symmetry and a diameter of ~27–30 nm. The viral capsid is composed of 180 copies of VP1 structured in 90 dimers. The VP1 can be divided in two regions, the shell (S) domain and the protruding (P) domain. The P domain can be further subdivided in the P1 and P2 being the P2 a big insertion in the P1 domain [15]. NoV genetic material is composed by a ~7.7 Kb positive sense, single-stranded, poly-adenylated RNA molecule [16]. The viral RNA carries three ORFs; the ORF1 codes for a poly-protein that suffers post-translational processing being transformed into 7 nonstructural proteins (NS1–NS7) [17]. The ORF2 codes for the main structural protein VP1 of 530 aa (~60 KD) [18], while the ORF3 codes for a highly divergent, small, basic structural protein VP2 of 212 aa (~22 KD) [19]. *Norovirus* is a genus within the *Caliciviridae* family that is also composed by 4 other genera: *Sapovirus*, *Lagovirus*, *Vesivirus* and *Nebovirus* [8]. The genus *Norovirus* is further divided into six genogroups (GI to GVI) and each genogroup can be divided in several genotypes. The hNoV are placed in three genogroups: GI, GII and GIV [20]. Most of the human isolates belong to genogroups GI and GII that are further subdivided in 31 genotypes (GI.1–9 and GII.1–22) [20], being genotype GII.4 the most prevalent in humans [21].

## 2. Host Genetics: The Role of Glycobiology in Mediating Enteric Virus/Host Interactions

**Figure 1:**
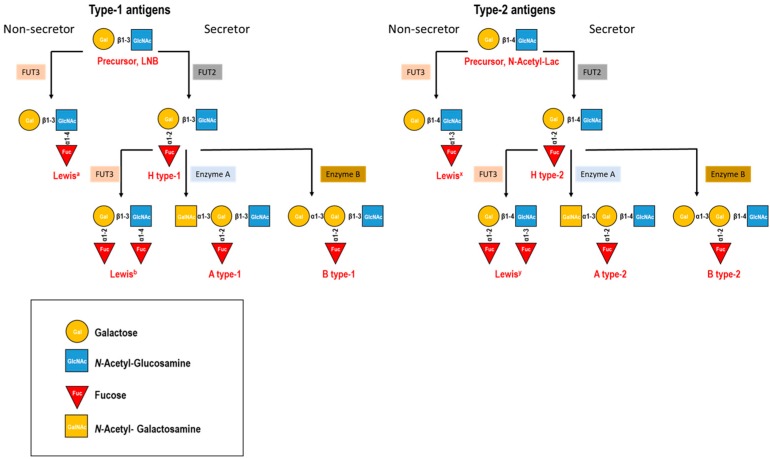
Biosynthesis routes and schematic structure of histo-blood group antigens (HBGAs) implicated in viral susceptibility. The type-1 (Lacto-N-Biose, LNB) and the type-2 (*N*-Acetyl-lactosamine, *N*-Acetyl-Lac) precursors are further elongated by the fucosyltransferase-2 (FUT2) and FUT3 enzymes to produce the H and Lewis antigens, as well as by the A and B enzymes to produce the A and B blood groups.

Several studies have associated hNoV and RV susceptibility to human histo-blood group antigens (HBGA), namely with the secretor status associated to the presence of at least one functional FUT2 (fucosyltransferase-2) allele, and with Lewis antigens (Le^a^ and Le^b^), determined by the FUT3 gene [22,23,24,25]. H and Lewis antigens, dependent on the FUT2 and FUT3 gene products activities, are oligosaccharide compounds made of *N*-acetyl-glucosamine, galactose, and fucose. The type-1 (galactose-β-(1→3)-*N*-acetyl-glucosamine, lacto-*N*-biose) and the type-2 (galactose-β-(1→4)-*N*-acetyl-glucosamine, *N*-acetyl-lactosamine) precursor disaccharides serve as substrate for the FUT2 enzyme that attaches a fucose residue to the galactose molecule via an α-(1→2) linkage, producing the H type-1 or H type-2 antigens, respectively (Figure 1). The type-1 and type-2 precursors are also substrates of the FUT3 enzyme that attaches fucose to the *N*-acetyl-glucosamine moiety via an α-(1→4) linkage in type-1 precursor or via an α-(1→3) linkage in type-2 precursor to produce Le^a^ and Le^x^ antigens, respectively (Figure 1). When the substrate is the H antigen the activity of the FUT3 enzyme produces Le^b^ (type-1) and Le^y^ (type-2) antigens (Figure 1). Furthermore, the H antigens can be further modified by the A and/or B enzymes in the epithelium to produce the A and/or B blood groups (Figure 1). Glycoconjugates of these HBGA on cellular surfaces are believed to participate in the first steps of viral infection, acting as receptors which are differentially recognized by distinct viral genotypes. Several interactions between NoV and the HBGA have been studied by enzyme linked immunosorben assay (ELISA) or haemagglutination-based assays using saliva, human milk, red blood cells or synthetic oligosaccharides as HBGA sources. In these assays several hNoV surrogates are usually employed, such as recombinant virus-like particles (VLP), self-assembled VP1 devoid of genetic material, or P-particles, consisting of oligomeric VP1 P-domains. A number of typical binding profiles have been described [26]. Thus, antigens H, A and O are known to bind the Norwalk GI.1 strain. VA387, a GII.4 strain, recognizes all ABO antigens, and VA207, a GII.9 genotype strain, recognizes Le^x^ antigen. Interaction of NoV with HBGA has also been demonstrated by structural analysis using X-ray crystallography. The significance of NoV interactions with HBGA on host susceptibility has been demonstrated by human volunteer challenge studies with the Norwalk GI.1 virus [23,27] and a GII.4 NoV strain [28,29]. Human susceptibility to NoV and its correlation to the HBGA geno- and phenotypes have also been studied in NoV outbreaks and in blood donors [30,31,32]. All together, these studies suggest that HBGA may function as viral receptors and play an important role as a host susceptibility factor for NoV. In addition to HBGAs other ligands such as heparan sulfate, citrate and sialic acid possess the ability to bind hNoV and may act as co-receptors during viral infection [33]. Diverse interactions between HBGA and RV have also been described. Recombinant VP8*, the protruding portion of RV VP4 protein, of P[8], P[4] and P[6] genotypes recognize the secretor HBGA. P[8] and P[4] are closely related genetically and both genotypes bind the Le^b^ and H-type 1 antigens [34]. P[6], a slightly further related genotype, binds the H-type 1 antigen only [34]. These binding specificities have been confirmed by haemagglutination of red blood cells, blocking by monoclonal antibodies, and binding of the complete virions. In addition, P[9], P[14] and P[25] genotypes bound specifically to the type A antigens [24,35], whereas P[11] interacted with single and repeated *N*-acetyl-lactosamine, the type-2 precursor glycan [36]. Direct evidence of RV-HBGA interaction has been shown by X-ray crystallography of a P[14] VP8* in complex with the type A oligosaccharide [24]. Based on these findings, human susceptibility to RV infections also relies on HBGA phenotypes. Several studies have suggested that the non-secretor phenotype (individuals with two null FUT2 alleles) was restrictive to P[8] and P[4] RV genotype infections, as revealed in analyses of symptomatic infections [35,37,38] or specific serum IgG levels [39]. Hence, FUT2 and Lewis polymorphisms could explain the low efficacy of RV vaccines in certain African populations, where the predominant viral strains and FUT2 and Lewis genotypes differ from Western populations [38].

## 3. Host Genetics: The Role of Glycobiology in Mediating Microbiota/Host Interactions

The niche where the enteric viruses RV and NoV replicate, the gastrointestinal tract, is inhabited by trillions of commensal bacteria. It constitutes a complex ecosystem where the microbial components play relevant roles in host physiology and the imbalances in its composition, referred to as dysbiosis, have been linked to certain disease conditions [40,41]. Similar to the influence of HBGA on viral infectivity, it is being discovered that host glycobiology affects gut microbial composition. Intestinal microbiota feeds on nutrients derived from the diet but it is also specialized in obtaining carbon and energy from host glycans present at mucosal surfaces and gastrointestinal tract-adapted bacteria possess a repertory of specialized enzymes (glycosyl hydrolases and carbohydrate transporters) for their metabolism. As an example, l-fucose occurs at relevant concentrations at the mammalian gastrointestinal tract in epithelial surfaces and in mucosal secretions as part of fucosylated glycans, including HBGA, and it is released as a free sugar by the action of microbial α-l-fucosidases, being an important carbohydrate for microbial intestinal physiology [42]. Host fucosylation in germ-free animals is low and it is induced by microbial colonization. It has been discovered that fucosylation can be activated by signals triggered by intestinal bacterial commensals such as *Bacteroides thethaiotaomicron* [43]. Fucosylation is also important in certain disease states. After a systemic infection, a rapid intestinal fucosylation mediated by MyD88-sensing of bacterial products and stimulation of FUT2 expression occurs in mice. This in turns supports the growth and activity of commensal beneficial bacteria, and helps in maintaining the host–microbiota symbiosis [44]. For some pathogenic *E. coli*, exposure to l-fucose reduces the expression of virulence genes (the locus of enterocyte effacement of enterohemorrhagic *E. coli* (EHEC) [45]), and studies with isogenic strains demonstrated a reduced fitness at the gastrointestinal tract in mutants unable to use l-fucose [46,47]. By the contrary, under certain circumstances l-fucose metabolism may favor pathogen dissemination [48]. Similar to the role of fucosylated HBGAs in RV and NoV attachment, these glycans can function as bacterial receptors for mucosal attachment of pathogens as well as commensal bacteria. Bacterial pathogens that bind to these sugars and fimbria recognizing HBGA have been described [49]. Also, commensal and beneficial bacteria such as *Lactobacillus* express lectin activities able to bind HBGA [50].

In addition to these examples with particular bacteria, the potential role of mucosal fucosylated HBGA on shaping the global intestinal microbiota composition has been addressed by studying the impact of the secretor phenotype (FUT2) on microbial diversity and abundance of specific microbial taxa by 16S rDNA sequencing from stool samples. Several studies including humans from different geographical locations and mouse models with humanized microbiota determined that non-secretor individuals (unable to synthesize H-antigen structures: fucose-α-(1→2)-galactose-β-(1→3/4)-*N*-acetyl-glucosamine) display a less diverse bacterial population [51,52,53] and several bacterial types, such as species of the genus *Bacteroides* and the *Lachnospiraceae* family, were more abundant in non-secretor mice and humans [51,54]. Notwithstanding, some *Bacteroides* species (*B. plebeius* and *B. fragilis*) were increased in secretor-positive individuals in other studies [51]. The non-secretor phenotype resulted in diminished *Bifidobacterium* species [55], whereas additional works showed an increase of the *Prevotellaceae* and *Paraprevotellaceae* taxons for this group [54]. These variations in the results may derive from the fact that, with only one exception, the number of analyzed subjects has been generally low (*n* = 18 to 39) and the analyses may not be exempt of the occurrence of confounders (e.g., gender, nutritional habits), which limits the reliability of the results. In a study carried out with a large cohort (1500 twins), no differences in the intestinal microbiota could be evidenced between secretor and non-secretors individuals. Even in this large study results might be biased, as the analyzed subjects were 90% women with an average age of 61 years and the study excluded from the analyses the microbial taxa that were present in less than 10% of the samples. This leaves open the possibility that less abundant microbial taxa may vary depending on the secretor status. Furthermore, the possibility exists that specific physiological conditions related to age, diet or health status modulate the impact of the secretor phenotype on the microbiota. In this sense, the differences seen in the microbiota composition in wild-type mice versus *Fut2−/−* mice disappeared when animals followed a diet depleted of polysaccharides [53]. Also, a FUT2 effect on the microbiota composition was evidenced in a human cohort (*n* = 47, including 29 Crohn’s disease patients and 18 healthy controls) only when the inflammatory bowel disease variable was introduced in the analyses, which allowed a clear separation of secretor phenotypes by their microbiota composition [52] and also in an study carried out with pregnant women (*n* = 123, 15 non-secretors) [56], which reinforces the idea that the FUT2 effect may only became evident under certain circumstances. In conclusion, studies with larger cohorts are still needed to obtain more robust results on the effect of the secretor status on the microbiota. In addition, the origin of the reported differences based on FUT2 still has to be established. These differences may present nontrivial causes as a result of the competence for nutritional resources and complex cross-feeding and other ecological relationships that are established within the different members of the intestinal microbiota [57].

## 4. Intestinal Microbiota and Susceptibility to RV and NoV Infections: Lessons From In Vitro and Animal Models

**Figure 2:**
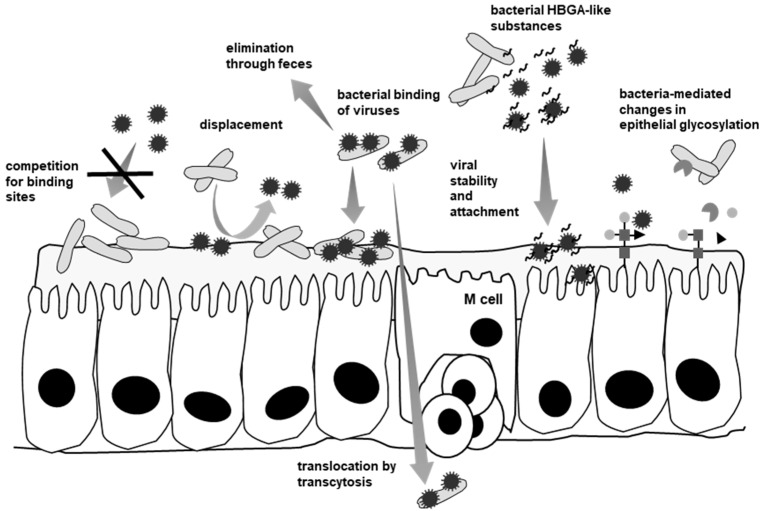
Proposed interactions of commensal intestinal bacteria with enteric viruses and their effects on viral accessibility and attachment to target cells. Physical interactions of bacteria with enteric viruses can promote or block viral infectivity. The binding of viral particles by bacteria can promote their elimination in the feces, stimulate their attachment to the mucosa or their transcytosis. Alternatively, bacteria can block viral binding sites or modify the glycosylation state of the mucosa, which in turn affects viral attachment. Bacterial components released to the lumen (lipopolysaccharide (LPS) or HBGA-like carbohydrates) have been found to stabilize the virions and enhance their attachment to cells.

**Figure 3:**
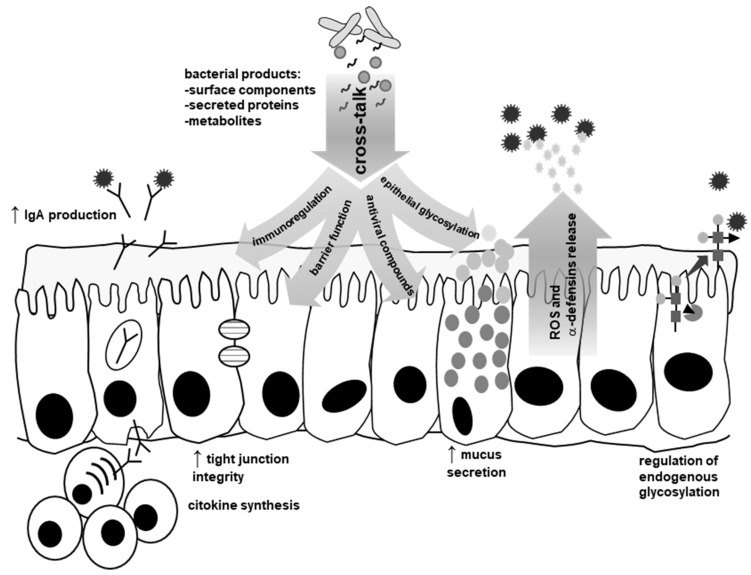
Effects on viral infectivity triggered by the cross-talk probiotic–microbiota–host. Bacteria produce diverse molecules that participate in a cross-talk with epithelial or immune cells. This cross-talk triggers diverse mechanisms that impact viral infectivity. Immunoregulation (enhanced production of specific sIgA, cytokines such as IFN-γ or IFN-β or regulation of lymphocyte populations) elicited by bacteria can limit viral infection. The cross-talk enhances the barrier function and induces the synthesis of molecules that can reduce infectivity: mucins, reactive oxygen species (ROS) or certain defensins. At the same time, host mucosal glycosylation is regulated by the endogenous microbiota.

Intestinal probiotics such as *Lactobacillus rhamnosus* GG, *Bifidobacterium lactis* Bb-12 or the yeast *Saccharomyces boulardii*, among others, have been thoroughly studied for their beneficial effects on the incidence and severity of viral diarrhea [58,59]. Physical interactions with RV and NoV and with components of the mucosal surface that are targets for viral binding have been proposed as mechanisms for physical blocking of virus attachment (Figure 2). Additionally, immunoregulation and reinforcement of the intestinal barrier as a consequence of the cross-talk that is established between commensal and probiotic bacteria and the epithelial and immune cells have arisen as a major mechanism mediating the antiviral effects of the microbiota (Figure 3). Secretion of molecules that interfere with viruses, such as increased mucus production or the synthesis of potential antiviral compounds (e.g., reactive oxygen species and some type of defensins) have also been reported to be regulated by the enteric microbiota (Figure 3) and it is known that the glycosylation status of the intestinal mucosa can be influenced by commensal microorganisms [43,60].

Probiotic bacteria (*L. rhamnosus* GG and *B. lactis* Bb-12) are able to bind RV at their surface (the calf RV NCDV strain and human Wa strain) at different levels and this has been proposed as a way for interfering with viral infection by sequestering viral particles and promoting their elimination through feces [61]. Experiments with hNoV (GII.3, GII.4, GII.6 and GII.7 genotypes) have shown that the enteric bacteria *Enterobacter* sp. strain SENG-6, an intestinal Gram-negative isolate, is able to bind the viral particles at its surface due to the presence of HBGA-like molecules of the A, B and H types that form part of extracellular secreted polymers [62]. hNoV surrogates (P-particles of GI.1 and GII.4 genotypes) also demonstrated their capacity to interact with the surface of Gram-positive and Gram-negative enteric commensals and probiotics [63], and binding of hNoV VLP (GI.6 and GII.4) has been evidenced for human intestinal isolates belonging to *Lactobacillus*, *Enterococcus*, *Bacteroides*, *Klebsiella*, *Citrobacter* and *Hafnia* [64]. When analyzing the effect of the bacteria/virus interaction on binding to cultured cells by using the hNoV P-particles model, it was observed that co-incubation of GI.1 hNoV P-particles with bacteria decreased their attachment to HT-29 cells. By the contrary, exclusion or displacement experiments of hNoV P-particles by the bacteria resulted in an increased binding of the subviral particles to cultures cells (up to 400% increase) [63]. This was in accordance with the observation that some probiotics enhanced the attachment of bacterial pathogens to cultured cells [65] and suggests that in some cases bacterial surface binding of hNoV may be promoting viral infection rather than limiting it.

Contrarily to the accepted role of the intestinal commensal microbiota and particular bacteria (probiotics) as a line of defense against enteric pathogens (colonization resistance), the microbiota has recently appeared as a third player in viral infectivity. Early evidence for a role of the intestinal microbiota in replication of viruses in mammals were obtained by using mice models with depleted intestinal microbiota (germ-free mice or animals treated with antibiotic cocktails) and viruses that do not target the gastrointestinal tract or do not cause AGE. The Murine Mammary Tumor Virus (MMTV) uses the microbiota for evading the immune system making use of a mechanism that induces tolerance to the resident microbiota. MMTV virions were discovered to interact with bacterial-derived lipopolysaccharide (LPS), triggering a TLR4-mediated response which results in immunosuppression [66]. Leukemia induced by the Murine Leukemian Virus (MuLV) was decreased in germ-free animals and it was hypothesized that the microbiota stimulated the increase in lymphoid cells, which are the target for MuLV [67]. Similar to MMTV, the intestinal-replicating poliovirus was seen to bind the polysaccharide moiety of LPS, which results in enhanced heat stability and capacity to bind to the poliovirus receptor in transgenic mice [68,69]. As a consequence, infectivity was reduced in germ-free or antibiotic-treated mice. The same effect was observed for the enteric reovirus T3SA+, which belongs to the same family than RV, suggesting that this may be an extended phenomenon in other enteric viruses [68]. A recent work employing a battery of intestinal microorganisms from the Gram-positive and Gram-negative groups (36 bacterial strains) showed that most of them displayed poliovirus binding capacity on their surfaces [70]. The highest percentage of binding was found for *Lactobacillus johnsonii* FI9785 (a poultry isolate), whereas the highest increase of poliovirus infection in HeLa cells was observed after co-incubation with *L. johnsonii* human fecal isolates. Although some strains enhanced viral infection, this enhancement did not correlate with the ability to bind poliovirus. By the contrary, poliovirus infection was linked to the capacity of the bacteria to bind to HeLa host cells [70]. An analogous situation was found when analyzing hNoV P-particles binding to HT-29 cells, where *Lactobacillus casei* BL23 and *E. coli* Nissle 1917, two strains that attach to the HT-29 surface, promoted the retention of P-particles on the cells surface [63].

During the last years, evidence of a positive role of the microbiota in enteric viruses infectivity have also been obtained for RV and NoV. While several cellular types are susceptible to RV infection in vitro, for hNoV it was not until recently that in vitro replication in human B lymphocytes was reported [71]. This in vitro infection required the intestinal microbiota, as was evidenced when infection was carried out with hNoV stocks (GII.4, isolated from feces) that were not filtered for eliminating the accompanying bacteria. While trying to dissect this microbiota-dependent infection, it was observed that particular bacteria such as *Enterobacter cloacae* ATCC PTA-3882, which expressed on its surface polymeric substances resembling H-type HBGA, enhanced virus attachment and infection in lymphocytes [71]. The same effect was obtained when purified H-antigen was used alone. Furthermore, in analogy to poliovirus, binding of hNoV (GI.1 and GII.4) to A- and B-like HBGA carbohydrates at the surface of *E. coli* LMG8223 and *E. coli* LFMFP861, respectively, enhanced its stability towards heat treatments [72]. Finally, reinforcing the in vitro data, microbiota depletion by using antibiotics reduced infectivity of murine NoV in mice [71] and prevented its persistent infection in a process that was dependent of the IFN-λ receptor [73]. A similar situation was observed for RV infection (murine RV strain EC) in an animal model, where antibiotic treatment reduced viral infectivity and enhanced serum and mucosal antibody response to RV [74]. However, in vivo experiments in the gnotobiotic pig model with *E. cloacae* ATCC 13047, showed that, in opposition to the in vitro results, this bacterium reduced hNoV (GII.4) shedding and that B cells were not the infection target [75]. Furthermore, a recent in vitro system for hNoV infection without the participation of the microbiota has been set up based on organoids derived from intestinal stem cells [14,76], for which there is still a profound debate on the strict requisite of the microbiota for hNoV infection and the cellular tropism of hNoV.

The underlying mechanisms on how the microbiota promotes RV and NoV viral infection are far from being understood and it is unknown whether binding to HBGA-coated bacteria or free HBGA participates in the entry process during infection, helping viruses to perform a productive attachment or it just serves the viruses to reach their infection sites. It has also been proposed that virus-loaded bacteria can be transcytosed by M-cells [77], allowing them to reach their infection targets. In addition, other direct or indirect effects can be expected (Figure 2). In any case, it becomes clear that certain intestinal viruses make use of the microbiota to modulate some steps of their infection process.

## 5. Intestinal Microbiota and Susceptibility to RV and NoV in Humans

The gut is a very complex ecosystem where several interplays are established between the host, the resident microbiota and pathogens responsible for AGE. The evolution of intestinal viruses in the gut ecosystem led to the establishment of interactions between viruses and the microbiota that are exploited by the pathogen to modulate some aspect of the infection process. Imbalances in the gut microbiota composition (dysbiosis) have been associated with increased risk of suffering bacterial intestinal infections (e.g., *Clostridium difficile* colitis [78]). Intestinal dysbiosis has also been modeled in pigs with humanized microbiota in the context of RV infection [79]. Pigs were intestinally colonized with the microbiota from feces of a healthy child and a child with a high degree of enteropathy, which is characterized by intestinal inflammation, increased permeability and microbial dysbiosis. After vaccination and RV challenge, the pigs with the ‘healthy’ microbiota exhibited an increased rotavirus-specific T-cell response and lower incidence or diarrhea [79]. This supports the idea that microbiota composition affects enteric virus infection. Many recent studies have also looked at the composition of the intestinal microbiota as a regulator of enteric virus susceptibility in human trials. The extended use of RV vaccines (oral vaccines composed of live attenuated RV strains) as a practice for diminishing AGE in children provides an excellent opportunity to link viral infectivity to the composition of the intestinal microbiota. In particular, the striking lack of efficacy of RV vaccine in low-income settings and particular geographical locations in terms of low vaccine take (trigger of specific antibodies) has been attributed to many possible causes, which include changes in the microbiota due to particular diets, health or nutritional status that may impact the microbiota [80].

Several studies have been conducted in African and Asian populations, which are characterized by a low response to the RV vaccines. When the fecal microbiota of children in Ghana receiving a RV vaccine (Rotarix^TM^) was analyzed (*n* = 78 with 39 non-responders), it was found that the microbiota on non-responders differed substantially from that of RV vaccine responders, with a number or *Prevotella*, *Bacteroides, Ruminococcus* and *Streptococcus* species linked to the non-responder group. In this case, the bacterial populations of responders were more similar to that of a Western population (Dutch cohort of age-matched individuals) that showed a good RV vaccine response [81]. Similar to the study with Ghanaian children, in another study conducted in Pakistan only 10 (15%) of infants responded to the RV vaccine (Rotarix^TM^) from a group of 66 children. The intestinal microbiota of these 10 responders was compared to that of a matched group of 10 non-responders, showing a correlation between the response to RV vaccine and the gut microbiota [82]. In this case, an increased ratio (2.6-fold) of Gram-negative versus Gram-positive bacteria was observed in RV vaccine responders as compared to non-responders. Also RV vaccine responders had higher ratios of *Firmicutes* and specific genera of the *Clostridium* cluster XI and *Proteobacteria* phylum (e.g., *E. coli* and *Serratia*). When compared to a Dutch children cohort of responders (156 individuals or 10 matched individuals) it was observed that this group also had a higher abundance of the *Proteobacteria* phylum with the class *Gammaproteobacteria* being especially abundant (15-fold higher with more abundant genera/species such as *Serratia*, *E. coli*, *Klebsiella*, and *Enterobacter*). However, other studies performed in India (Rotarix^TM^; *n* = 170, with 85 non-responders) showed no correlation between the gut microbiota and the efficacy of RV vaccine, in contrast to other factors such as the occurrence of distinct intestinal pathogens (e.g., the presence enteroaggregative *E. coli* was higher in RV vaccine responders) or the co-administration of the oral poliovirus vaccine [83]. While some authors speculated on the role of LPS released by *E. coli* and *Proteobacteria* in boosting the RV vaccine response [82]; clearly, more studies on the impact of the microbiota on the efficacy of the RV vaccines are needed. In this respect, prospective studies across different countries (India, Malawi and the UK) with standardized experimental designs are underway [84].

Unfortunately, the conducted studies did not analyze a likely effect of the secretor status on the vaccination outcome and, due to the lack of a vaccine, this kind of studies cannot be performed for hNoV. Knowing the association between the HBGA (secretor status and ABO blood groups) and the microbiota, it might be possible that the host glycobiology modulates the replication of the RV and hNoV by a direct interaction with these viruses and by an indirect effect mediated by the microbiota. This possibility was addressed by analyzing the levels of RV- and hNoV-specific IgA in saliva, the FUT2 genotypes and the intestinal microbiomes in a group of healthy adults (*n* = 35). Higher salivary anti-NoV and anti-RV IgA levels were related to the secretor status, indicating that this parameter may be used as an indicator of viral susceptibility. Single variable analyses showed that the overall microbial composition did not differ between secretors and non-secretors. Interestingly, when multiple variable analyses were applied, the incorporation of the anti-RV and anti-NoV IgA titers resulted in significant differences, in microbial composition, evidencing an interplay between the secretor status, the intestinal microbiota and viral susceptibility. During this study, associations were found between distinct bacterial taxa and viral susceptibility (measured as IgA titers against RV and hNoV) that were also detected in the RV vaccine trials. Thus, the increased numbers of *Bacteroidetes* that were linked to the non-secretor status were also found in non-responders from the Ghana RV vaccine assay [81]. Also, members of the *Ruminococcaceae* family were more abundant in Ghanaian non-responders [81] and correlated with lower RV and hNoV IgA titers in adults [54]. Positive and negative correlations were also found for some bacterial species and IgA titers to RV and hNoV. Thus, the levels of *Faecalibacterium prausnitzii* negatively correlated to hNoV IgA titers, whereas the levels *Akkermansia muciniphila* were related to increased levels of anti-RV IgA in adults [54]. Interestingly, members of the *Verrucomicrobiae*, to which *A. muciniphila* belongs, were also high in Dutch infants showing response to the RV vaccine [82]. These potential biomarkers of infectivity need to be further confirmed and, especially for RV, cohort studies involving infants under five years of age are necessary to study the interplay between secretor status, microbiota and viral infections.

## 6. Conclusions and Perspectives

The intestinal microbiota has emerged as a new key player in enteric virus infection. The discovered opposite roles (antagonist and enhancer of viral infection) probably reflects the complex interplays established in the intestinal niche, where viral infectivity, host mucosal glycosylation, and the microbiota are interconnected. Immunomodulation, virus–bacteria physical interaction, and the exploitation of bacterial products for enhancing stability, attachment, and viral entry are the most plausible mechanisms by which bacteria influence viral infection. The bacteria–risk correlations of RV/NoV infection are being discovered, even if the source for analyzing the microbial composition have been fecal samples, which are informative about the colonic microbiota but do not necessarily reflect the bacteria present at the jejunum and ileum in the small intestine where infection by RV and NoV occurs. Recent studies have shown that the human jejunum contains 10^3^ to 10^6^ bacteria per mL of intestinal content, consisting of the most abundant bacterial genera *Streptococcus*, *Prevotella*, *Veillonella* and *Fusobacterium* [85]. Interestingly, some relevant bacterial groups with possible implications in viral replication, such as members of the genera *Bacteroides, Lactobacillus* and *Ruminococcus,* are found in the ileum (10^7^ to 10^8^ bacteria per mL of ileal content) [86]. This prompts to the need for new mechanistic data that sustain the effect of particular microbial groups in the RV and NoV infection process and for discriminating between direct and indirect effects. Research in the enteric viruses and their interactions with the microbiota will probably pave the way for the development of new microbiota-targeting antiviral therapies. Many possibilities exist, including the use of bacteria with enhancing properties for the development of more effective oral vaccines (e.g., RV vaccines) or the modulation of the intestinal microbiota through different interventions (e.g., dietary interventions [87]) aimed at selecting a microbiota more prone to restrict RV or NoV replication.
